# Practical aspects of protein co-evolution

**DOI:** 10.3389/fcell.2014.00014

**Published:** 2014-04-22

**Authors:** David Ochoa, Florencio Pazos

**Affiliations:** ^1^European Molecular Biology Laboratory, European Bioinformatics Institute (EMBL-EBI)Hinxton, UK; ^2^Computational Systems Biology Group, National Centre for Biotechnology (CNB-CSIC)Madrid, Spain

**Keywords:** protein interactions, co-evolution, biological networks, interactome, mirrortree

## Abstract

Co-evolution is a fundamental aspect of Evolutionary Theory. At the molecular level, co-evolutionary linkages between protein families have been used as indicators of protein interactions and functional relationships from long ago. Due to the complexity of the problem and the amount of genomic data required for these approaches to achieve good performances, it took a relatively long time from the appearance of the first ideas and concepts to the quotidian application of these approaches and their incorporation to the standard toolboxes of bioinformaticians and molecular biologists. Today, these methodologies are mature (both in terms of performance and usability/implementation), and the genomic information that feeds them large enough to allow their general application. This review tries to summarize the current landscape of co-evolution-based methodologies, with a strong emphasis on describing interesting cases where their application to important biological systems, alone or in combination with other computational and experimental approaches, allowed getting new insight into these.

## Introduction

It is difficult to fully understand evolutionary phenomena without taking into account the important role played by co-evolution. Co-evolution, which can be defined as the interdependence between the evolutionary changes of two entities, plays an important role at all biological levels, from ecosystems to molecules. Co-evolution was first described at the species level. C. Darwin himself described the entangled evolution of orchids and their pollinators, in the sense that the length of the proboscis of the latest was related to the size of the orchid's corolla (Darwin, 1862). In the first half of the XX century, other biologists continued studying this phenomenon and establishing its genetic basis (Dobzhansky, 1950). The term “co-evolution” was originally coined by P. Ehrlich, who studied this phenomenon at the species level (Ehrlich and Raven, 1964). The definition of co-evolution as “reciprocal evolutionary change in interacting species” (Thompson, 1994) is the most accepted one today. From these early works, the idea of “interaction” becomes intimately associated to co-evolution. Co-evolution takes place between related or interacting entities and that is actually the reason for its utility at the molecular level.

The study of co-evolution at the molecular level is much more recent (Juan et al., 2013). At this level, co-evolution is evident between protein residues, so that in many cases changes (mutations) in positions related by functional or structural (i.e., space closeness) reasons are correlated. The practical utility of this observation is the prediction of residue contacts in protein structures, using sequence information as the only input (Juan et al., 2013). Going up in the “molecular hierarchy,” co-evolution is also evident between interacting and functionally related proteins. Many pairs of interacting proteins show entangled evolutionary histories. Such evolutionary entanglement can lead, in the extreme, to the disappearance of one of the proteins when the other is lost. This extreme phenomena is reflected in related patterns of presence/absence of the two proteins in a set of genomes, which is actually the basis of the “phylogenetic profiling” methodology for detecting interacting proteins (Pellegrini et al., 1999). In other cases, the evolutionary entanglement of interacting proteins is reflected in similar evolutionary histories but without reaching the extreme of the co-disappearance of the proteins. Since protein evolutionary histories are represented by phylogenetic trees, a common way of inferring protein co-evolution is by quantifying the similarity of the phylogenetic trees of the corresponding families (Pazos and Valencia, 2001). Such idea was inspired by observations at the species level: it was described that the phylogenetic trees of interacting species (e.g., parasites and their hosts or predators and preys) were similar, reflecting a process of co-adaptation between them. Back to the protein level, it was shown that there is a consistent relationship between tree similarity and interaction (physical or functional) of the corresponding proteins. That observation led to a large family of methodologies that predict protein interactions based on similarity of phylogenetic trees, using only sequence information as input.

Although co-evolution-based methodologies continue to be developed and improved, they reached a point at which they are mature enough to be used by the community and form part of the standard toolbox of computational methods used by Molecular Biologists. Not only because their performances, both in terms of accuracy and coverage, increased in the last years, but also because they are now implemented in usable software and web interfaces. The aim of this review is to provide an overview of the current landscape of the main co-evolution-based methodologies, including recent examples of their application to different biological systems.

## Co-evolutionary approaches

**Figure 1:**
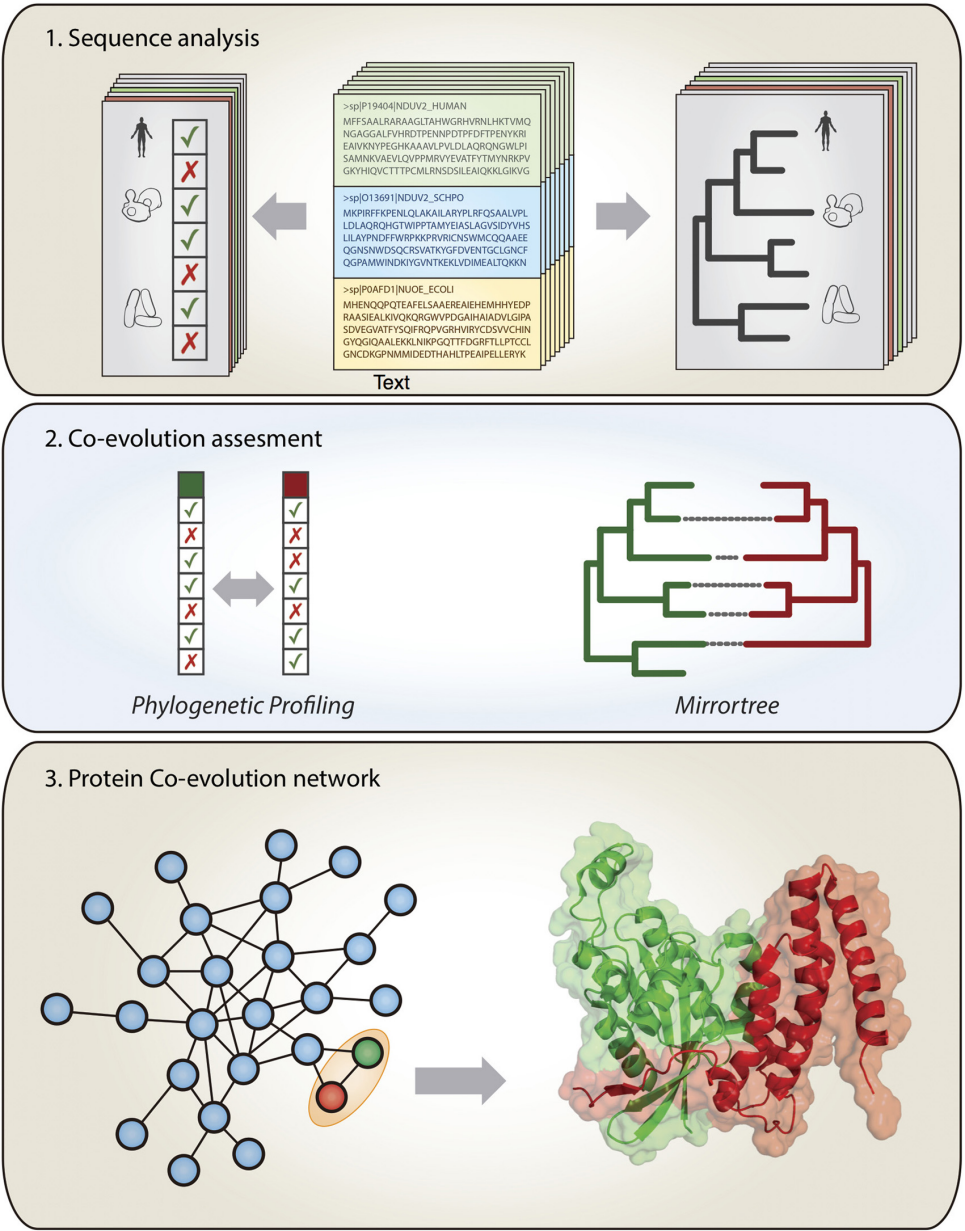
**Extracting co-evolutionary linkages from genomic information to study protein interactions**. **Top panel**: the large amount of available sequence and genomic information is used to construct phylogenetic profiles (patterns of presence/absence of the genes in a set of organisms) and phylogenetic trees at a genome-wide scale. **Middle panel**: Co-evolving pairs of proteins (green and red) are detected by the similarity of their phylogenetic patterns and/or the similarity of their phylogenetic trees. **Bottom panel**: the co-evolutionary linkages obtained in this way contain a lot of information on the interactions and functional relationships for the proteins in the organisms of interest.

The evolutionary forces entangling interacting proteins very often restrict their sequence evolution to the point of being perceivable at a genomic level. In a time governed by the “omics” techniques, a family of computational methods aim to detect the marks left on the genome by co-evolving molecules as a symptom of interaction (Shoemaker and Panchenko, 2007; Juan et al., 2013). The associations detected do not necessarily imply physical interaction, but can also reflect involvement in similar biological functions, such as the same protein complex, the same metabolic pathway or the same operon. In this section, we review the different computational methods of co-evolutionary basis, focusing on their application scope and potential limitations (Figure 1).

### Phylogenetic profiling

Methods based on phylogenetic profiles rely on the observation that functionally associated and potentially interacting proteins evolve in a codependent manner tending to be jointly inherited or eliminated. This extreme case of co-evolution between functionally related genes has been explained as a consequence of “reductive evolution,” where the loss of one of the members of the cooperative interaction dismisses the evolutionary pressure to maintain its partner. Alternatively, the recruitment of a new protein requires the acquisition of its partner to form the new functional complex. As a consequence of this phenomenon, the patterns of presence/absence of the two interacting partners in a set of genomes would tend to be similar.

A phylogenetic profile summarizes that pattern of presence/absence of a given gene in a set of reference organisms. At first, the profiles were encoded as binary representations, where “1” denotes the presence of an ortholog gene in a given organism, and “0” its absence (Gaasterland and Ragan, 1998; Marcotte et al., 1999; Pellegrini et al., 1999). Changes in the information contained on the profiles lead to a number of variations of the original phylogenetic profiling approach. For instance, instead of being binary, the profiles can contain quantitative information, such as the similarity of the ortholog with that in a reference organism (Date and Marcotte, 2003). Other profiles successfully encoded phenotypic traits to predict functional linkages (Levesque et al., 2003; Gonzalez and Zimmer, 2008). On the other hand, although the phylogenetic profiles were originally designed to contain information at full-sequence level, profiles based on domain presence/absence successfully predicted domain interactions (Pagel et al., 2004; Ranea et al., 2007).

Profile-profile similarity has been calculated using different metrics such as euclidean distance (Marcotte et al., 1999), mutual information (Date and Marcotte, 2003) or Hamming distance (Wu et al., 2003).

Besides profile similarity, functional linkage has also been observed between pairs of anti-correlated profiles encoding for pairs of genes excluding each other (Morett et al., 2003). In a similar way, higher order relationships described by logic operators have been explored in order to look for complementation and other functional relationships relating triplets of profiles (Bowers et al., 2004b). Another interesting phenomenon evident in the phylogenetic profiles of some pairs of interacting proteins is the “disrupted co-occurrence” (the presence of A implies that of B but not the other way around). These cases can point to asymmetric protein relationships (A needs B but B does not need A) and as such provide additional functional information to static interactions (Notebaart et al., 2009; Schneider et al., 2013).

A comprehensive list of pre-calculated similarities between protein phylogenetic profiles can be found in resources such as STRING (Von Mering et al., 2003), Prolinks (Bowers et al., 2004a) or ECID (Andres Leon et al., 2009).

From a practical perspective, one of the most critical issues on phylogenetic profiling methodologies is the selection of the reference set of organisms. The number of completely sequenced genomes continues growing. Nevertheless, the best predictions are not always obtained with profiles based on all the available genomic sequences (Sun et al., 2007). Indeed, the accumulation of close organisms, as well as the taxonomic bias in the sequenced genomes affect the profiles, decreasing their performance in interaction prediction. Moreover, depending on the type of functional relationship the prediction is aimed at, the optimal set of organisms might change. Profiles based on organisms belonging to the three super-kingdoms display better performances for detecting conserved interactions, whereas species in the same superkingdom are more accurate for pathways (Jothi et al., 2007). More systematic approaches using 565 bacterial genomes confirmed that sub-samples of organisms can achieve better performances than the whole set of available genomes (Muley and Ranjan, 2012). In order to automatically select the reference set of organisms, recent studies use machine-learning algorithms trained with known sets of interactions to improve the accuracies of the arbitrary selection of organisms (Simonsen et al., 2012).

From an evolutionary perspective, the presence/absence of every gene in a phylogenetic profile is equally weighted, independently of the number of potential evolutionary events needed to explain it. The number of potential gene gains/losses might be informative in order to estimate the statistical likelihood of a similarity score. As a matter of chance, similarities based on multiple evolutionary events will be more reliable than those with the same score but based on a fewer number of events. The idea of combining phylogenetic profiles with phylogenetic trees to weight the gene co-presence or co-absence is exploited in different studies by using Markov models (Barker and Pagel, 2005; Cohen et al., 2012), kernel trees (Vert, 2002) or explicit comparisons (Zhou et al., 2006). Another limitation arises from the fact that some gene clusters might strongly co-evolve in some parts of the evolutionary tree, while exhibiting a weak co-dependency in other organisms. This non-homogenous distribution of the co-evolution, referred as “local co-evolutionary” problem, has been subject of different studies, but remains as a computationally challenging task (Kim and Subramaniam, 2006; Tuller et al., 2010).

Besides the previously mentioned limitations, some technical issues have to be addressed when comparing phylogenetic profiles. This methodology requires complete and well-annotated genomes to be sure of the existence or absence of a given gene. Even in those cases, the orthology assignment is not trivial, being particularly critical in eukaryotes, where the presence of multiple domains, pseudogenes or inactivated genes difficult the proper assignment. Furthermore, essential proteins or those specific of a given organism can not be addressed by this approach as they are encoded as flat profiles. In summary, this methodology displays optimal results when analyzing gene pairs with clear orthologs uniformly distributed on the tree of life and presenting a reasonable number of common gain/loss events.

### Similarity of phylogenetic trees

Phylogenetic profiles are based on genomic landmarks left by dramatic events affecting whole genes or genomic regions (genes gains and loses). However, that approach ignores subtle changes on the sequences of interacting proteins, which might be also reflecting co-evolution. Such coordinated sequence changes might shape the phylogenetic trees of interacting proteins increasing their similarities. The first observations of this phenomenon qualitatively described that the phylogenetic trees of some pairs of interacting families were more similar than expected (Fryxell, 1996; Pages et al., 1997). Despite not being quantified or assessed in an exhaustive way, the similarity between the phylogenetic trees of those protein families was interpreted as a symptom of protein co-evolution.

The first method to quantify tree similarities calculated the correlation of the distance matrices as descriptors of the phylogenetic trees. The algorithm was soon scaled up to predict protein interactions at a genome-wide scale based on similarities of automatically generated phylogenetic trees (Pazos and Valencia, 2001). This approach, generically termed *mirrortree*, uses a simple pipeline to evaluate the eventual interaction between a pair of proteins. On its initial implementation, for the two protein families for which co-evolution is to be evaluated, multiple sequence alignments are generated aligning all the orthologs present in a set of reference genomes. Phylogenetic trees for each of the protein families are generated from the multiple sequence alignments, frequently using fast and simple algorithms such as neighbor-joining. Finally, tree similarities are estimated by calculating the correlation coefficient between equivalent inter-ortholog distances in the two alignments. Consequently, unambiguous correspondence between the sequences of the two alignments is required, in order to allow the distances in both trees to be compared. This problem is normally solved by selecting one single ortholog per organism, leading to a natural mapping between the leaves of both trees, given by the organisms. Alternative solutions try to match the equivalent orthologs under the hypothesis that the correct mapping maximizes the tree similarity (Ramani and Marcotte, 2003; Izarzugaza et al., 2006, 2008; Tillier et al., 2006; Hajirasouliha et al., 2012). Other modifications of the original mirrortree algorithm suggest that when cophenetic distances are extracted from the branch lengths of the phylogenetic trees the prediction performance becomes slightly improved (Pazos et al., 2005).

That pipeline is now implemented in the Mirrortree server, which provides a user-friendly web interface to allow non-expert users to overcome most of the aforementioned tasks (Ochoa and Pazos, 2010) and to interactively and graphically inspect the tree similarity. The server combines a powerful and automatic pipeline for tree reconstruction with an interactive interface to explore tree similarities. In the simplest case, the user can provide single sequences as input, although more advance users can provide their own alignments or even trees, and tune the parameters of the workflow.

One of the main limitations of the original mirrortree algorithm is the large number of false positives produced as a consequence of the unspecific tree similarities. One of the possible reasons for such a large amount of highly correlated trees between unrelated proteins can be due to the background tree similarity occurred as a consequence of the speciation events. Since the proteins under study are both affected by the ongoing speciation process, we expect both trees to display certain basal similarity, independently of their eventual interaction. The correction of that unspecific similarity due to the underlying speciation process (shared by both trees and the tree of life) is addressed by different methods using different statistical corrections and different representations of that background similarity. The first attempts used the 16SrRNA tree as a representation of the speciation process and tried to subtract its phylogenetic distances directly from the distance matrices of the interacting candidates (Pazos et al., 2005; Sato et al., 2005). The corrected methodology, renamed *tol-mirrortree*, obtained higher performances than the original *mirrortree*. More successful examples of co-evolution detection on ligand-receptor interactions have been reported, this time applying a background speciation correction (Tiwary et al., 2009). However, these corrections are incomplete in the sense that they consider each value in the distance matrix as independent, which is not the case for phylogenetic trees. If we change a given distance on the tree, the lengths of all other paths involving the modified branch should also be changed to adapt to the new distance. Some sophisticated methods try to consider the distance dependency problem by aligning high-dimensional embeddings of the trees (Choi and Gomez, 2009). Instead of using canonical trees to remove unspecific similarities, other methods use the tendencies obtained from large collections of protein families as an evidence of the background similarity. One of the first attempts to take advantage of this contextual information introduced the partial correlation coefficient as a measure of similarity. This metric calculates the correlation between a pair of phylogenetic vectors, excluding the information of a third vector containing the background information. By using the variability of the phylogenetic data as third vector, the prediction false positive rate was drastically reduced (Sato et al., 2006). ContextMirror, an alternative method that also uses contextual information to reduce the background similarity, goes one step further: the unspecific signal associated to a pair of phylogenetic trees can be removed by comparing them with many others (Juan et al., 2008). As a preliminary step, this method calculates the pairwise *mirrortree* correlation coefficients between all the proteins in a given organism. In the resulting matrix of tree similarities, a coevolutionary profile is defined as the vector of correlation coefficients of a given protein with the rest of the proteome. The correlation between coevolutionary profiles is calculated as an estimate of how similar are the co-evolutionary patterns of both with the rest of the proteome. Alternatively, partial correlation between coevolutionary profiles reports the correlation of a pair of coevolutionary profiles when a third coevolutionary profile is taken into consideration. ContextMirror amazingly reduces the number of false positives, producing performances comparable to some experimental techniques (Juan et al., 2008).

The similarity of the phylogenetic trees, likewise the similarity of the phylogenetic profiles, is greatly influenced by the reference set of organisms used to generate the trees. In practical terms, disregarding technical issues such as the computational power required for generating and comparing trees based on all available genomes, two factors have to be considered when selecting the reference set: the problem of redundant organisms and the type of interactions intended to detect. As a consequence of the “non-uniform” sequencing efforts, the trees generated with all the sequenced genomes available nowadays contain a large bias toward the organisms of interest, for instance containing dozens of strains of some model bacteria. The *mirrortree* algorithm, far from benefiting from the new information, is severely affected by such genomic redundancy (Herman et al., 2011). As a consequence, independent studies suggest that the interaction prediction is improved when the organism redundancy has been removed. Those studies also suggest that the redundancy problem is partially overcome by some of the methods that remove background similarities, such as the correlation of coevolutionary profiles and ContextMirror (Herman et al., 2011); or tol-mirrortree (Muley and Ranjan, 2012). On the other hand, the type of interaction to be detected constrains the selection of organisms. Certain subsets of organisms seem to be more suitable for predicting certain types of interactions. This result makes sense in the light of the phylogenetic distribution of the organisms and the nature of the predicted interactions. Local tree similarities involving close homologs are more likely to be related with recent interactions, whereas global similarities of the phylogenetic trees may evidence a co-evolution occurring since ancestral species (Herman et al., 2011). Supporting evidence suggest that mirrortree predictions normalized by the level of conservation (evolutionary span) of the candidate interaction significantly improve the interaction predictions (Zhou and Jakobsson, 2013). Dealing with this non-homogenous nature of the co-evolutionary signal is not trivial as it raises certain combinatorial problems when trying to evaluate the similarity locally in all possible subsets of tree clades. A particularly successful method, MatrixMatchMaker (MMM), approaches this problem by looking for the largest common submatrix compatible with the evolutionary distance matrices under comparison (Tillier and Charlebois, 2009). MMM changes the paradigm of phylogenetic tree comparison by reducing the problem to the minimal common submatrix. The evolutionary span of the protein interaction is no longer relevant as the method dynamically adapts to maximize tree similarity. As a desired side effect, the method tolerates matrices including paralogs, since these will most likely be excluded from the final similarity if wrongly assigned. Although a recent implementation reduces the computationally expensive task of optimizing matrix similarity (Rodionov et al., 2011), this algorithm still demands a significant amount of resources when working with large number of sequences.

Co-evolution has also been observed at the residue level, as pairs of individual protein positions which are close in 3D or related in some way and tend to mutate in a coordinated fashion (see Juan et al., 2013, for a review). Consequently, a number of methodologies try to infer co-evolution between two proteins based on the “accumulation” of co-evolutionary signals between their corresponding residues (Pazos and Valencia, 2002; Yeang and Haussler, 2007; Burger and Van Nimwegen, 2008). This evidence of co-evolution at sub-protein levels also led some authors to study whether restricting the assessment of co-evolution to certain subsets of protein residues might increase the performance of the methods or provide additional information on the interactions. In most cases, these restrictions were based on structural criteria (surfaces, structural domains, etc), when such information is available. For instance, by comparing the domains of the alpha and beta subunits of the mithocondrial F1-ATP synthetase, seven pairs of domains that are known to interact present higher correlations than the two non-interacting pairs (Jothi et al., 2006). As when comparing full sequence proteins, these predictions improve their performance when removing the background similarity of the phylogenetic trees. Indeed, the predictions are more accurate when the background removal is applied to the trees based on the most conserved residues, indicating that both signals are more easily disentangled on those regions (Kann et al., 2007). The presence of regions that not necessarily share the evolutionary constraints of the whole protein has also been tested on protein interfaces with contradictory results. Studies suggest that residues in the interfaces of stable interactions evolve at a relatively slow rate, consequently affecting the eventual co-evolutionary signal with their interacting partners. In contrast, residues involved in transient interactions would present a higher plasticity, leaving little or no co-evolutionary signal in the interaction interfaces (Mintseris and Weng, 2005). In both cases, the residues not present in the interface still contain enough co-evolutionary signal to predict the interaction (Kann et al., 2009). These results have been interpreted as a clear symptom that the co-evolutionary signal is uniformly distributed along the protein sequence showing no improvement by limiting the study to either the protein surface or the interaction interface (Hakes et al., 2007). Others reported a stronger co-evolutionary signal on the interfaces (including a structural neighborhood) than in the same number of randomly selected residues outside the binding neighborhood (Kann et al., 2009). These analyses were based on limited and not necessarily overlapping sets of structures, so the true extent of their conclusions is hard to evaluate. On the other hand, phylogenetic trees based on residues predicted as accessible have been shown to be more informative for predicting physical protein interactions (Ochoa et al., 2013). Structural information is necessary and critical in order to fully understand interactions at the molecular level, nevertheless the definition of a general recipe on how to incorporate it in co-evolution-based methods remains elusive.

## Examples of applications

In this section we describe some examples of recent applications of co-evolution-based approaches to different biological problems. In principle, these methodologies can be applied to any organism, and indeed different groups used them to predict interactions in species covering the whole range of taxonomical diversity, from bacteria (Juan et al., 2008), to fungi (Clark et al., 2011) and human (Havugimana et al., 2012). The successful application to eukaryotic organisms is more recent since in those the (automatic) generation of accurate multiple sequence alignments and trees, key for applying these methodologies, has some additional difficulties compared with prokarya (location of orthologs, multidomain proteins,…).

Strong co-evolutionary signals are found in pairs of families where one of them has to accommodate its evolutionary rate to that of the other, accelerated for some reason. For example, the nuclear-encoded components of the NADH-ubiquinone reductase complex show such accelerated evolutionary rate to accommodate the intrinsically fast evolution of their mitochondrial-encoded counterparts. This results in evolutionary entanglements that can be used to predict interactions between these two sets of proteins, interactions that were latter confirmed experimentally (Gershoni et al., 2010). Co-evolution was also found between the mitochondrial-encoded rRNAs of the mitochondrial ribosomes and the nuclear-encoded proteins of these organelles (Barreto and Burton, 2013). A similar strategy based tree-similarity was used to study the co-evolution between the nuclear- and chloroplast-encoded members of the RuBisCO protein complex (Pei et al., 2013).

A link between to apparently independent processes such as redox homeostasis and cellular timekeeping was found based on the presence of co-evolutionary signals (Ochoa and Pazos, 2010) between pairs of proteins of these processes (Edgar et al., 2012). That was complemented with experimental observations on the oxidation/reduction cycles of peroxiredoxin being universal markers of circadian rhythms in bacteria, eukaryotes and archaea, despite the huge mechanistical differences of these processes in the three superkingdoms (Edgar et al., 2012).

Recently, Havugimana et al. (2012) generated a large catalog of human soluble protein complexes combining experimental mass spectrometry with computational inference of interactions using, among others, the MMM tree-similarity-based co-evolutionary approach (Tillier and Charlebois, 2009). That approach had been previously used alone to obtain a human co-evolutionary network that was shown to reflect protein physical interactions (Tillier and Charlebois, 2009; Bezginov et al., 2012). In another interesting combination of experimental and computational approaches, Lu et al. filtered the intrinsically noisy Hi-C data on “contacts” between chromosome regions using co-evolutionary information so as to obtain a reliable prediction of the target genes for distal regulatory elements (DRE) in human (Lu et al., 2013). In this case, they applied phylogenetic profiling to the presence/absence patterns of DREs and genes. Gene phylogenetic profiling was also recently used to generate a network of relationships between human proteins useful, among other things, to locate disease-related modules (Tabach et al., 2013).

Co-evolution is also especially evident in systems were the interaction patterns have to maintain interactions while continue evolving to acquire new functions and/or avoid crosstalk with the ancestral systems. This is the case of signaling cascades, where a paralogous expansion has to rapidly diverge to avoid interference with the original system, and such change has to be compensated by the interacting partners so as to maintain a functional cascade. In this sense strong co-evolutionary signals were found, for example between members of the bacterial two-component signaling system (Capra et al., 2012). Molecular systems related to sex are another prototypic case of rapidly evolving systems where co-evolution plays an important role, since they have to differentiate and acquire specificity quickly so to avoid cross-fertilization, while maintaining the specific interactions at the same time. In *Brassica campestris*, sequencing of 14 alleles allowed to find co-evolution between the (male) SCR and the (female) S receptor kinase (Watanabe et al., 2000). This system is involved in the pollen discrimination mechanism. Similarly, deep-sequencing was recently used to study the co-evolution between male and female fertilization proteins of abalone snails (Clark et al., 2009). Similar cases were found in Yeast. For example Zamir et al. found that the proliferating cell nuclear antigen (PCNA) co-evolves with its interaction partners across the whole fungi phylogeny, what contributes to generate hybrid incompatibility and promoting speciation (Zamir et al., 2012).

Transcription factors were also shown to co-evolve with their DNA-binding sites so as to maintain interactions while continue diverging (Kuo et al., 2010; Yang et al., 2011).

## Conclusions

From the first anecdotic qualitative observations of tree similarity for some pairs of related protein families (e.g., insulins and their receptors Fryxell, 1996) a lot of efforts were devoted to better understand the phenomenon of protein-protein co-evolution and to find practical ways of taking advantage of it. The genomic revolution allowed the genome-wide generation of multiple sequence alignments and trees so as to study the extent of this phenomenon and statistically assess its relationship with protein interactions (Pazos and Valencia, 2001). From that point onwards, variations of the original idea and new methodologies were developed so as to achieve higher accuracies and coverages in protein interaction prediction (see Juan et al., 2013). These methodological improvements, together with user-oriented implementations of these methods and usable web interfaces (e.g., Ochoa and Pazos, 2010) took us to a point where we can say that these approaches are mature enough to form part of the toolboxes of bioinformaticians and molecular biologists. And, as such, they are currently being used, alone or in combination with other computational and experimental approaches, for getting insight into important biological systems. Even if we still have a long way ahead in terms of improving these methodologies, they reach the required performance for being applied in a quotidian basis.

Co-evolution-based approaches, together with other computational approaches which also use sequence and genomic information for inferring protein linkages, form a family of approaches termed “context-based methods” (Harrington et al., 2008; Wass et al., 2011), which complement the classical homology-based methods in obtaining information on different aspects of the proteins from their raw sequences (Von Mering et al., 2003).

Co-evolution is not yet a completely understood phenomenon. Getting insight into its ultimate causes will contribute to the improvement of the methodologies. For example, it is not totally clear whether the observed co-evolution between interacting proteins is due to a process of specific co-adaptation or to more unspecific causes which could be “pushing” the evolutionary rates of the two proteins in a similar magnitude (Pazos and Valencia, 2008).

What is clear by the discussed examples and others is that the ultimate reason for the observed co-evolution seems to be allowing the two (interacting/related) partners to evolve and change while maintaining the interaction. An alternative way to maintain the interaction is to stay conserved, and indeed that is the case for some interactions. But in most interactions the partners have to evolve for one reason or another. In some cases this evolution is mainly “neutral,” for example intrinsic rapid evolution due to lack of repair mechanisms in the genomes of eukaryotic organelles of bacterial origin, such as the mitochondrial and chloroplast examples commented. In these cases, the nuclear-encoded interactors of these proteins have to accommodate their evolutionary rates, and such co-evolutionary signal can be detected. In other cases one of the interactors simply changes to acquire new functions and loses the previous ones (to avoid crosstalk), and consequently the partner has to change too so as to maintain a functional complex. Again, we find a parallelism here with co-evolution at the species level (see Introduction), since it is known that, at that level, co-evolution is also allowing species to change while maintaining ecological interactions such as mutualism (Thompson et al., 2013).

### Conflict of interest statement

The authors declare that the research was conducted in the absence of any commercial or financial relationships that could be construed as a potential conflict of interest.
